# How diet therapy affects obesity-associated depressive symptoms: from mechanism to therapeutics

**DOI:** 10.3389/fnins.2026.1846186

**Published:** 2026-07-10

**Authors:** Ruiqing Wang, Zhilin Chen, Yiwei Wang, Yangpu Zhang, Aiqun Song

**Affiliations:** 1 College of Acupuncture-Moxibustion and Orthopedics, Hubei University of Chinese Medicine, Wuhan, China; 2 Xinhua Hospital of Hubei University of Chinese Medicine, Wuhan, China; 3 Hubei Provincial Hospital of Integrated Chinese and Western Medicine, Wuhan, China; 4 Hubei Provincial Hospital of Traditional Chinese Medicine, Wuhan, China; 5 Hubei Shizhen Laboratory, Wuhan, China; 6 Affiliated Hospital of Hubei University of Chinese Medicine, Wuhan, China; 7 Hubei Provincial Clinical Research Center for Acupuncture and Moxibustion in Obesity Treatment, Wuhan, China

**Keywords:** depressive symptoms, diet therapy, neuroendocrine, neuroinflammation, obesity

## Abstract

Obesity stands as a formidable 21st-century public health crisis, with its capacity to aggravate depressive symptoms gaining increasing clinical attention. Traditional treatment models often treat these two conditions separately. However, recent research evidence suggests a complex network linking obesity and depressive symptoms across metabolism, behavior, and mental health, with dietary patterns proposed as a key upstream modulator of both metabolic and psychological pathways. The specific mechanisms by which diet influences obesity and depressive symptoms remain unclear. Therefore, this narrative review focuses on analyzing molecular connections between diet, obesity, and depressive symptoms, including adipose tissue inflammation, the gut-brain axis, the hypothalamus-pituitary–adrenal axis, insulin and brain-derived neurotrophic factor levels, and neuroplasticity. We discuss the possible pathways and effects of different diet therapies in regulating metabolism and simultaneously impacting mental health, including calorie restriction diet, intermittent fasting, ketogenic diet, low glycemic index diet, plant-based diet, Mediterranean diet, Dietary Approaches to Stop Hypertension, among others. This review aims to provide a scientific basis for precision nutrition and personalized, sustainable diet therapies in clinical practice, promoting awareness and improving treatment strategies for depressive symptoms in obese patients.

## 1 Introduction

Driven by shifting global lifestyles, obesity has emerged as a major public health challenge worldwide (Lingvay et al., 2024). The number of obese individuals is projected to reach approximately 1 billion by 2030, posing a significant health economic burden globally (Nagi et al., 2024). The clinical picture is further complicated by the frequent co-occurrence of obesity with insulin resistance, type 2 diabetes, coronary heart disease, stroke, and with depression being a significant mental health-related complication (Blasco et al., 2020). Depression is one of the most common neuropsychiatric disorders in clinical practice (GBD 2019 Diseases and Injuries Collaborators, 2020). A recent systematic review showed that individuals with a body mass index (BMI) ≥ 30 kg/m^2^ have approximately a 55% higher risk of developing depression than those with normal BMI (Luppino et al., 2010). As BMI further increases, the effect size between obesity and depression also increases, clearly establishing their link (Menniti et al., 2025). This dual burden not only increases morbidity in affected individuals but also substantially complicates clinical management strategies.

Obesity and depression have been traditionally managed within the separate domains of endocrinology and psychiatry (Oracz et al., 2025). However, this fragmented treatment model may lead to the neglect of the shared pathophysiological mechanisms between the two diseases, thus affecting actual treatment outcomes (Lv et al., 2022). Currently, increasing clinical and experimental evidence is challenging this dichotomy (Keswani et al., 2025). Furthermore, conventional drugs for treating obesity, such as orlistat and semaglutide, may cause adverse reactions such as gastrointestinal dysfunction and pancreatitis, while commonly used antidepressants such as paroxetine and escitalopram can lead to metabolic disorders and some neuropsychiatric symptoms (Cao et al., 2024; Lincoff et al., 2023; Son and Kim, 2020). Diet therapy, on the other hand, has attracted much attention due to its gentle effects, ease of acceptance, long-term applicability, and ability to influence both metabolic and psychosomatic pathways (Adan et al., 2019).

Diet is the primary medium for the exchange of substances between the external environment and the human body. Besides serving as an energy source, diet also influences various physiological processes, including inflammatory states, endocrine signaling, microbial ecology, and neural plasticity (Mostafavi Abdolmaleky et al., 2025). This means that by rationally selecting and combining foods, we can simultaneously regulate multiple system pathways, including metabolic and psychosomatic pathways—a concept that diet therapy embodies (Waśkiewicz, 2026). Therefore, obesity-related depressive symptoms can, to some extent, be understood as a diet-sensitive metabolic-psychiatric comorbidity. A comprehensive and integrated understanding of the pathogenesis of both obesity and depressive symptoms, and the underlying mechanisms of diet therapy’s clinical effects, is crucial for future improvements in treatment plans and patient prognosis.

Previous reviews have summarized the effects of various dietary patterns on metabolism and psychosomatic health. A cross-species systematic review demonstrated that probiotic supplementation may attenuate high-fat diet-induced cognitive impairment and depressive-like behaviors through modulation of the gut–brain axis—a pathway that represents a shared mechanistic basis with several plant-based diet therapies examined in this review (Lof et al., 2022). Additionally, a narrative review of older adults found that higher dietary inflammatory index scores were associated with increased risk of depression and metabolic disorders (Yildirim and Sanlier, 2024). Some researchers have also revealed through meta-analysis that hypocaloric diets may alleviate depressive symptoms in patients with metabolic conditions such as obesity in the short term (Applewhite et al., 2024; Paris et al., 2024). However, none of these studies have provided a holistic analysis of the complex neuro-metabolic-immune network mechanisms involved in diet, obesity, and depressive symptoms, nor have they compared the differences between various diet therapies.

This review aims to integrate existing experimental and clinical evidence to explore the connections between various diet therapies and obesity and depressive symptoms, analyzing the underlying metabolic-psychological coupling mechanisms. This review also aims to elucidate the mechanistic basis by which diet therapy modulates the neuro-metabolic-immune network, offering a theoretical foundation for clinical application and future research directions.

## 2 Obesity and how it exacerbates depressive symptoms

Obesity is now a common metabolic disease characterized by excessive fat accumulation, systemic inflammation, and endocrine disorders (Xu et al., 2025). The core pathophysiological mechanisms of obesity encompass sustained positive energy balance and dysregulation of metabolic and neuroendocrine signaling pathways, compounded by genetic predisposition, hormonal dysregulation, and behavioral factors (Adolph and Tilg, 2024). Epidemiological and clinical intervention experiments consistently demonstrate that the mainstream Western diet, characterized by high saturated fat, high refined carbohydrates, and low fiber, is the main driver of the obesity epidemic (Hall et al., 2019). This dietary pattern bypasses the body’s satiety signals, overactivates the reward pathway of the limbic system in the brain, triggers chronic inflammation and appetite disorders, and traps the body in a vicious cycle of energy surplus, ultimately manifesting as a pathological increase in body fat percentage (Jia et al., 2025).

As mentioned earlier, the increased risk of depression due to obesity has been confirmed. Furthermore, meta-analysis has revealed a bidirectional association between obesity and depression, independent of confounding factors such as sociodemographic factors (Luppino et al., 2010). Genetic studies have revealed overlap in susceptibility gene loci such as FTO, NEGR1, DCC, RPL31P12, CADM2, PMAIP1, and PARK2 between obese and depressed patients, establishing a foundation for the pathophysiological link between the symptoms of the two diseases (Ding et al., 2022; Yuan et al., 2025). Therefore, obesity should be recognized as a multi-system disease involving metabolic disorders, endocrine imbalances, and neurological dysfunction, with depressive symptom being one of its important complications.

**Figure 1:**
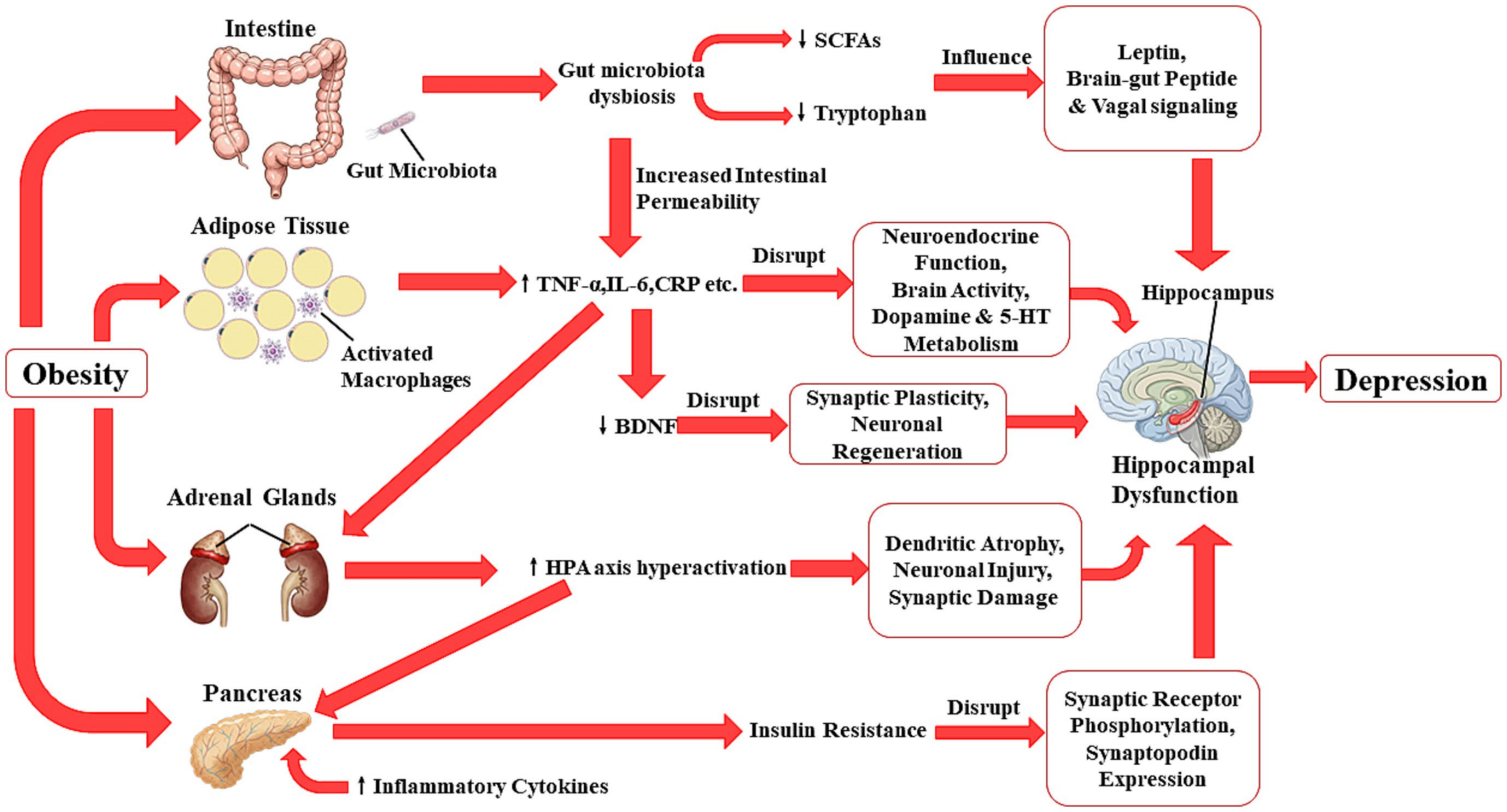
The pathophysiological mechanism by which obesity exacerbates depressive symptoms. In obese individuals, the gut microbiota is disrupted, which leads to reduced production of SCFAs (Short Chain Fatty Acids) and tryptophan absorption, disrupting leptin, brain-gut peptide secretion and vagal signaling. Simultaneously, impaired intestinal barrier function and adipose tissue hypertrophy cause upregulation of inflammatory factors such as TNF-α (Tumor Necrosis Factor-α), IL-6 (Interleukin-6), and CRP (C-Reactive Protein). This results in neuroendocrine dysfunction, affecting brain activity, dopamine and 5-HT (5-Hydroxytryptamine) metabolism. It can also lead to decreased BDNF (Brain-Derived Neurotrophic Factor) levels, impairing synaptic plasticity and neuronal regeneration. Increased inflammation also causes HPA axis hyperactivation, leading to dendritic atrophy, neuronal injury, and synaptic damage. Obesity-associated metabolic and inflammatory disturbances result in insulin resistance, which in turn leads to neuronal insulin receptor phosphorylation and reduced synaptopodin expression. These factors can all lead to dysfunction in the brain, especially the hippocampus, which in turn can cause depression.

Currently, researchers have investigated the molecular links between obesity and depressive symptoms, focusing on gut microbiota dysbiosis, adipose tissue inflammation, hormone levels, and glucose metabolism disorders. These links may become the main targets of diet therapy (Figure 1).

### 2.1 Adipose tissue inflammation

A recent study showed that in obese individuals, adipose tissue cells enlarge and the microenvironment is remodeled (Miranda et al., 2025). Increased mechanical stress can lead to the infiltration of immune cells, represented by lipid-associated macrophages (LAMs), and increased secretion of pro-inflammatory factors such as nicotinamide phosphoribosyltransferase (NAMPT), tumor necrosis factor-*α* (TNF-α), interleukin-6 (IL-6), and C-reactive protein (CRP). These peripheral inflammatory signals are transmitted to the brain via multiple pathways, including disruption of the blood–brain barrier, vagal afferent signaling, and peripheral immune cell infiltration, where they activate microglia and the indoleamine 2,3-dioxygenase (IDO) pathway (Miller and Raison, 2016; Tan et al., 2023). Once neuroinflammation is triggered, activated microglia further produce pro-inflammatory cytokines that impair serotonergic and dopaminergic neurotransmission, disrupt neurogenesis in the hippocampus, and promote glutamate excitotoxicity, collectively contributing to the development of depressive symptoms (Mostafavi Abdolmaleky et al., 2025). Intraperitoneal injection of TNF-α in mice increases the metabolism of dopamine and serotonin (5-HT) in the nucleus accumbens, thereby decreasing brain stimulation reward and inducing depression-related anhedonia (Figure 1) (van Heesch et al., 2013). Meanwhile, research shows that the use of anti-inflammatory drugs such as nonsteroidal anti-inflammatory drugs and cytokine inhibitors can reverse depressive states to some extent, further supporting this view (Xu et al., 2020).

### 2.2 Insulin resistance (IR)

IR is also a common pathophysiological state in obesity. Insulin not only regulates glucose metabolism but also plays a crucial role in the central nervous system. Insulin can cross the blood–brain barrier and modulate synaptic receptor expression and function by regulating excitatory and inhibitory receptor levels on the synaptic vesicle membrane, thereby regulating synaptic plasticity and influencing neural pathway function (Ohtsuki, 2024). In particular, insulin modulates the expression and membrane trafficking of AMPA and NMDA receptors at excitatory synapses, as well as GABA-A receptors at inhibitory synapses, thereby regulating synaptic excitability and plasticity critical for mood regulation and cognitive function (Pang and Han, 2012). Deficits in insulin receptor signaling have been linked to reduced BDNF expression and impaired activation of downstream signaling cascades, including the PI3K/Akt, which is a key mediator of neuronal survival and synaptic plasticity implicated in the pathophysiology of depression (Bansal et al., 2021). In the CA1 region of the rat hippocampus, insulin resistance significantly attenuates the phosphorylation of insulin receptors in response to exogenous insulin stimulation, while concurrently reducing the expression of synaptopodin, a protein essential for spine apparatus formation and synaptic plasticity (Figure 1). This results in impaired long-term potentiation in this region, disrupting hippocampal circuits involved in stress regulation and mood control (Scherer et al., 2021). The interference of IR with these physiological processes is likely a cause of abnormal appetite control, stress response, and mood regulation.

### 2.3 Gut-brain axis disorders

Chronic inflammation does not occur in isolation but is closely linked to alterations in gut microbial composition, which further amplify systemic inflammation and contribute to depression. Both obesity and depression are associated with gut microbiota imbalance caused by a decrease in the proportion of specific bacteria such as Bifidobacteria, Lactobacillus, and Akkermansia (Wang et al., 2025). Changes in the gut microbiota can affect the production of short-chain fatty acids(SCFAs) and tryptophan absorption, thereby altering the inflammatory state of multiple tissues, including nerves and fat, as well as brain-gut peptide secretion and vagus nerve signaling, ultimately affecting host metabolism and brain function (Figure 1) (Khan et al., 2026; Niu et al., 2024). Previous studies have shown that SCFAs can significantly affect leptin expression, and leptin acts on the hypothalamus across the blood–brain barrier to control appetite (Ferreira-Hermosillo et al., 2023). Furthermore, animal experiments have shown that leptin acts on the hippocampus and other regions to regulate synaptic plasticity, significantly shortening depressive-like behavior in mice during forced swimming tests (Chen et al., 2024). These findings suggest that altered gut microbiota ecology is a key mechanism connecting diet, metabolism, and emotional state.

### 2.4 Hypothalamic–pituitary–adrenal (HPA) axis dysfunction

The HPA axis is a crucial system regulating glucocorticoid production. Hyperfunction of the HPA axis has been observed in both obese and depressed patients, manifested as abnormal adrenocorticotropic hormone (ACTH) levels and altered responses to dexamethasone inhibition tests (Huang et al., 2024). A high-fat diet induces obesity in mice while simultaneously placing them in a state of chronic stress. In this state, HPA axis hyperfunction leads to excessively high glucocorticoid levels, further promoting visceral fat deposition and insulin resistance, while also causing hippocampal dendritic atrophy, neuronal loss, and synaptic lesions leading to mood disorders (Figure 1) (Selman et al., 2025). Additionally, HPA axis dysfunction may induce abnormal changes in the central 5-HT and dopamine systems, resulting in depressive states (Hodes et al., 2024). Persistent HPA axis activation and chronic glucocorticoid exposure also impair neuronal plasticity by suppressing BDNF expression.

### 2.5 Reduced levels of brain-derived neurotrophic factor (BDNF)

Impaired synaptic plasticity and reduced neuronal regeneration associated with decreased BDNF levels represent another critical mechanism linking obesity to central nervous system dysfunction and depressive symptoms. BDNF, produced by neurons and other cell types including endothelial cells, supports hippocampal neurogenesis and synaptic maintenance (Amadio et al., 2022). Low BDNF levels can inhibit the expression and phosphorylation of its downstream effector proteins, leading to decreased density of hippocampal dendritic spines and weakened long-term potentiation, resulting in impaired learning and cognitive function (Bahadorpour et al., 2023). Oxidative stress associated with obesity is accompanied by the generation of large amounts of reactive oxygen species (ROS), which increases DNA damage and further reduces BDNF levels (McLachlan et al., 2023). These metabolic disturbances may be important causes of mood disorders (Figure 1).

## 3 The impact of diet therapy on obesity and depressive symptoms

Modern diet therapy is a nutrition-based intervention strategy that primarily adjusts the quantity and quality of an individual’s diet to promote health (Semnani-Azad et al., 2025). Based on available experimental and clinical evidence, we summarize the potential effects of common dietary interventions on obesity-associated depressive symptoms.

### 3.1 Energy restriction diets

**Figure 2:**
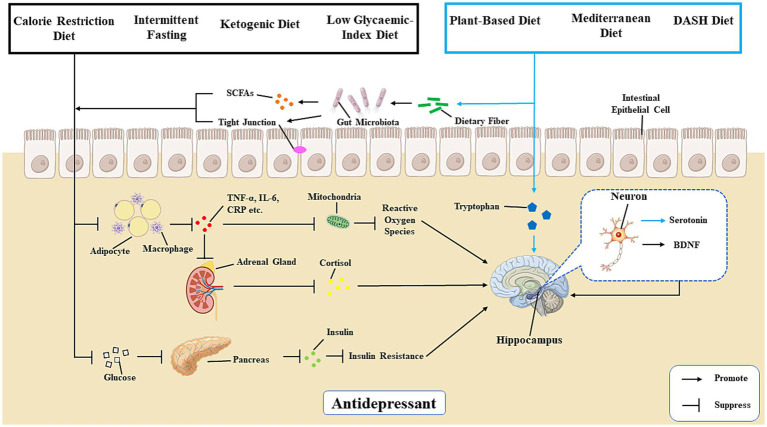
Common dietary therapies and how they affect depressive symptoms in obese patients. These dietary therapies achieve their effects of improving metabolism and producing antidepressant effects through diverse molecular mechanisms. Calorie restriction diet can reduce blood sugar fluctuations to alleviate insulin resistance, thereby improving neuronal biosynthesis. It can also improve the adipocyte microenvironment, reduce the release of inflammatory factors like Tumor Necrosis Factor-α (TNF-α), Interleukin-6 (IL-6), and C-Reactive Protein (CRP), and also alleviate oxidative stress. The ketogenic diet, in addition, promotes Brain-Derived Neurotrophic Factor (BDNF) synthesis. Furthermore, intermittent and low-glycemic index diet help regulate cortisol rhythms. Plant-based diet, Mediterranean diet, and Dietary Approaches to Stop Hypertension (DASH) are rich in tryptophan, which can directly promote serotonin biosynthesis. The dietary fiber in these diets can improve gut microbiota structure, promote Short Chain Fatty Acids (SCFAs) production and maintain tight junctions in intestinal epithelial cells, thereby reducing inflammation and oxidative stress.

Energy/calorie restriction diets are among the most widely studied dietary interventions (Paris et al., 2024). They typically involve reducing an individual’s energy intake by more than 30%, while maintaining normal intake of protein, vitamins, minerals, and water (Zhang et al., 2024). Energy restriction diets have long been a major intervention for obesity, significantly reshaping fat distribution and adjusting fasting insulin levels. A 12-month randomized controlled trial (RCT) involving 439 participants showed that participants receiving calorie restriction diet had lower Brief Symptom Inventory-18 depression scores. Specifically, the mean score decreased by 1.7 in the dietary therapy group and increased by 0.7 in the control group (*p* = 0.03) (Imayama et al., 2011). Current clinical trials often combine them with prebiotics and exercise therapy. Such interventions further reduce adipose tissue hypertrophy and improve insulin sensitivity. They also increase endorphin release, reduce oxidative stress, and regulate serotonin metabolism, thereby improving neuropsychiatric symptoms (Figure 2) (Reagan et al., 2021). However, a recent study has shown that strict calorie restriction can put the body in a state of stress (Wang et al., 2022). Under stress, excessively high levels of glucocorticoids may lead to neuronal damage and subsequently depression. Similarly, a clinical study has reported elevated depression scores in subjects after long-term strict calorie restriction, along with fatigue and decreased subjective alertness (Becker et al., 2023). The levels and duration of energy restriction vary across different studies, and these studies often combine different therapies or supplements (Wang et al., 2022). Conflicting results among these studies suggest that clinical optimization of energy restriction strategies may be needed to reduce the risk of depression.

### 3.2 Intermittent fasting

Unlike continuous energy restriction, intermittent fasting manipulates meal timing rather than solely reducing caloric intake, thereby inducing distinct metabolic adaptations. Intermittent fasting includes 5:2 intermittent fasting (5 days of normal eating, 2 days of calorie restriction per week) and time-restricted eating (eating within a specific time window each day, such as 8 h of eating followed by 16 h of fasting) (Varady et al., 2021). Studies have shown that intermittent fasting can lower insulin levels, blood pressure, inflammatory markers, and blood lipids, contributing to weight loss and improved metabolic health (Xie et al., 2024). Some studies suggest that regular intermittent fasting helps restore normal cortisol rhythms, rebalancing the HPA axis to reduce chronic stress (Baek et al., 2024). Animal studies have found that time-restricted eating increases hippocampal BDNF expression, promotes ketone body production, enhances autophagy, reduces oxidative stress biomarker malondialdehyde, and regulates circadian rhythm gene expression to protect neurological function and improve mood (Figure 2) (Abdel-Rahman et al., 2024). However, some researchers believe that some of the benefits of intermittent fasting mainly come from energy restriction, indicating that its efficacy requires further verification (Hansen et al., 2024).

### 3.3 Ketogenic diet (KD)

The KD is a low-carbohydrate, high-fat diet. The classic KD has a fat-to-protein to carbohydrate ratio of 4:1, with a recommended protein intake of less than 1 g/kg body weight. In recent years, researchers have developed more palatable diets with a higher protein-to-carbohydrate ratio, such as the medium-chain triglyceride diet and the modified Atkins diet. These all have similar effects, improving metabolism and reducing weight by inducing a shift in metabolism from carbohydrate utilization to ketone bodies produced from fat breakdown (Figure 2) (Chrysafi et al., 2024). However, high-quality randomized controlled clinical trials investigating the antidepressant mechanisms of KD are still limited. Animal studies have demonstrated that KD exerts multiple effects in regulating metabolism and improving depressive symptoms, including enhancement of mitochondrial biogenesis, reduction of ROS production, upregulation of BDNF expression, promotion of long-term neuronal potentiation, attenuation of neuroinflammatory responses, and altering post-translational modifications of proteins through lysine acetylation and *β*-hydroxybutyrylation (Li et al., 2020). KD pretreatment can also reduce the release of lipopolysaccharide (LPS)-induced pro-inflammatory factors in the blood and brain. Other reports indicate that β-hydroxybutyrate (BHB) produced by KD can increase brain levels of gamma-aminobutyric acid (GABA) and the GABA/glutamate ratio (Liang et al., 2025). What’s more, a RCT involving 415 participants showed that after 6 months of intervention, the KD group had a significantly lower Depression Anxiety Stress Scale-21(DASS-21) score compared to the control group (mean score = −2.0; 95%CI (−3.8 to −0.2); *p* = 0.033) (Bennell et al., 2022). However, recent studies have found that transplanting fecal microbiota from KD-treated patients into mice can induce anxiety-like behavior, suggesting that its molecular biological mechanisms require further investigation (Ito et al., 2024).

### 3.4 Low glycemic index (GI) diet

A low-GI diet emphasizes the intake of low-GI foods such as whole grains and legumes, while limiting high-GI foods such as refined carbohydrates and added sugars to reduce postprandial glycemic response (Toh et al., 2020). High-GI diets increase cortisol secretion, leading to obesity, while low-GI diets can help regulate weight by delaying gastric emptying, increasing satiety, stabilizing blood glucose levels, and reducing fluctuations in hunger-related hormones. A clinical study found a significant difference in the Center for Epidemiologic Studies Depression Scale(CES-D) score between the high-GI and low-GI diet groups, with an adjusted least-squares mean difference of 0.76 (SE = 0.31, *p* = 0.02) (Breymeyer et al., 2016). Furthermore, a clinical study found that a low-GI diet significantly reduced levels of oxidative stress markers in patients’ blood, including malondialdehyde (MDA), paraoxonase-1 (PON-1), total antioxidant status (TAS), and total oxidative status (TOS), and lowered depression scale scores (Depboylu et al., 2025). The reasons may include the effects of inhibiting blood glucose fluctuations on neurotransmitter synthesis, tissue inflammation, and oxidative stress (Figure 2). Clinical studies have shown that its effects are similar to those of energy-restricted diets (Pavithran et al., 2020). However, some low-GI diet studies have insufficient assessment of nutrients other than carbohydrates, and the required GI value for low-GI diets varies across different studies.

### 3.5 Plant-based diets

Plant-based diets emphasize a diet primarily composed of plant-based foods, categorizing foods into healthy plant-based foods, unhealthy plant-based foods, and animal-based foods. Researchers often assign a plant-based diet index based on the frequency of consumption of these foods by the subjects; the higher the frequency of consumption of healthy plant-based foods, the higher the index. Healthy plant-based foods have a lower energy density and higher fiber content, which helps with weight management (Wang et al., 2024). A cross-sectional study involving 1,949 middle- and older-aged adults found that greater adherence to the plant-based diet was significantly associated with fewer depressive symptoms, as assessed by the CES-D score (*β* = −0.095, 95% CI: −0.154 to −0.036; *p* = 0.002) (Millar et al., 2026). This may be because these foods are rich in antioxidants and polyphenols (Aleksandrova et al., 2021). Foods such as apples and onions contain dietary polyphenols, which, even in small doses, can reduce the excessive breakdown of monoamine neurotransmitters, thus exhibiting antidepressant activity (Wang et al., 2024). On the other hand, legumes and nuts are rich in tryptophan, which can control serotonin synthesis to inhibit the occurrence of depressive symptoms. Dietary fiber, abundant in plant-based foods, can be metabolized into SCFAs by gut microbiota, regulating immune cell function, enhancing intestinal barrier integrity, and improving gut microbiota composition, thereby reducing endotoxin translocation and systemic inflammation (Figure 2) (Dong and Cui, 2022). It is important to note that a plant-based diet does not require complete vegetarianism; an unbalanced plant-based diet can also affect neurological function. Some studies have found a higher risk of depression in vegetarians, possibly related to deficiencies in nutrients such as vitamin B12, iron, and omega-3 fatty acids. Therefore, some studies suggest adding foods like eggs and fish to a healthy plant-based diet (Zamani et al., 2020).

### 3.6 Mediterranean diet

The Mediterranean diet is rich in fruits, vegetables, nuts, legumes, and whole grains, low in fat and carbohydrates, and extremely low in processed foods. This dietary pattern is rich in antioxidants, polyphenolic compounds, omega-3 fatty acids, and dietary fiber, which can inhibit pro-inflammatory signaling pathways such as nuclear factor κB (NF-κB), reducing the production of pro-inflammatory cytokines in the gut and internal environment, thereby improving metabolism and mental state (Xiao Y. et al., 2024). Multiple RCTs have shown that the Mediterranean diet can increase satiety, improve insulin sensitivity, and regulate gut microbiota, effectively reducing visceral fat accumulation and promoting weight loss (Khavandegar et al., 2024). Large RCTs have also demonstrated significant reductions in depressive symptoms in subjects with high adherence to the Mediterranean diet. Recent comparative studies suggest that adherence to the Mediterranean diet is associated with greater reductions in depression scale scores than the ketogenic diet (Mela et al., 2026). The Mediterranean diet can improve gut microbiota composition through dietary fiber, increasing the proportions of Faecalibacterium, Eubacterium, Lactobacillus, and Bifidobacterium. The SCFAs produced by these bacteria can protect the brain and regulate inflammation by inhibiting histone deacetylases, thereby inducing Treg cell development and producing an antidepressant effect (Yamada et al., 2011). Olive oil, a representative ingredient in the Mediterranean diet, has antioxidant properties and can increase *Δ*-9 desaturase activity, thus maintaining neuronal cell membrane homeostasis and reducing the interference of inflammatory factors on endothelial cell BDNF secretion. Furthermore, the Mediterranean diet is rich in vitamins B6, B12, and folic acid, which serve as essential cofactors in the conversion of dietary tryptophan into serotonin and other monoamine neurotransmitters, thereby contributing to potential antidepressant effects (Figure 2) (Stewart, 2018).

### 3.7 Dietary approaches to stop hypertension (DASH)

The DASH diet was originally designed to prevent hypertension, emphasizing the intake of fruits, vegetables, whole grains, and low-fat dairy products while limiting saturated fat and sodium. It is characterized by low calories, high fiber, rich nutrients, and a strong feeling of satiety, thus aiding in weight management (Daneshzad et al., 2022). Cohort studies have found that adherence to the DASH diet can also have a certain preventive and therapeutic effect on depressive symptoms (Gianfredi et al., 2021). Furthermore, a 12-week RCT also showed that adherence to the MIND diet significantly improved DASS depression scores in overweight and obese women, and the improvement remained significant after adjustment for potential confounders (*β* = −2.64, 95% CI: −5.05 to −0.23; *p* = 0.033) (Golmohammadi et al., 2025). This may be because the composition of the DASH diet is similar to that of the Mediterranean diet and plant-based diets, improving mood through the attenuation of oxidative stress and reduction of systemic inflammation (Khavandegar et al., 2024). The plant-based foods such as grains and vegetables abundant in the DASH diet also contain considerable amounts of dietary magnesium and folic acid, which can reduce inflammation by lowering C-reactive protein levels and play a role in various brain responses. They also help improve glucocorticoid receptor function and restore HPA axis homeostasis (Figure 2). Simultaneously, reduced fat and carbohydrate intake can also increase endorphin levels in the brain and improve sleep quality (Arab et al., 2022). Based on the DASH diet, dietary strategies such as the MIND (Mediterranean-DASH diet intervention to delay neurodegenerative diseases) diet, which combines with the Mediterranean diet, have been developed, further enhancing its neuroprotective effects (Golmohammadi et al., 2025).

### 3.8 Dietary supplements and others

Besides various systemic dietary adjustments, people frequently use various dietary supplements. The mechanisms of action of these substances are mostly similar to the aforementioned diet therapies. For example, some probiotic supplements can promote SCFA production and regulate the HPA axis, while magnesium supplements can regulate cellular metabolism and reduce the production of inflammatory factors such as IL-6, TNF-*α*, and interferon-*γ* (Abiri et al., 2022). These supplements can also inhibit the activation of IDO, thereby diverting tryptophan metabolism away from the kynurenine pathway and toward the serotonin synthesis pathway, ultimately increasing 5-HT availability in the central nervous system. Other supplements, such as vitamin D, participate in 5-HT synthesis, and Ghana seed extract is rich in 5-HT precursors (Akhondzadeh et al., 2020). Supplementation with specific probiotic strains such as Lactobacillus and Bifidobacterium can directly upregulate the synthesis of 5-HT or its precursor 5-hydroxytryptophan, affecting activity in mood-related brain regions (Rondanelli et al., 2012). However, part of the mechanism of dietary supplements and dietary patterns remain unclear.

### 3.9 Comparison of clinical efficacy of different diet therapies on depressive symptoms in obese patients

We have introduced the commonly used diet therapies in clinical practice above, and their effects on regulating depressive symptoms in obese patients have been supported by some experimental and clinical evidence. However, whether the clinical efficacy of these different diet therapies on depressive symptoms in obese patients is the same remains to be verified. Currently, there are few existing clinical studies comparing the efficacy differences between different diet therapies, and their intervention periods are usually short, lacking long-term efficacy assessments (Table 1). Taken together, the Mediterranean and MIND diets, which emphasize natural foods, high fiber, and antioxidants, have a more significant short-term effect on mood improvement compared to other diets. On the other hand, the KD diet, characterized by a high fat and high protein ratio, is not satisfactory in improving depressive symptoms while improving obesity symptoms. While a simple energy restriction diet can improve the mood of subjects to some extent, it may need to be combined with other dietary supplements or changes in nutritional composition to achieve better efficacy. Studies comparing the efficacy of other diet therapies are also scarce. The significant effects of the Mediterranean diet and similar approaches may lie in their positive impact on multiple metabolic-psychological pathways, including gut microbiota, insulin sensitivity, amino acid balance, anti-inflammatory effects, and anti-oxidative stress. In contrast, the strict carbohydrate restriction in KD may significantly increase dietary stress in patients. Prolonged lack of carbohydrate intake, including grains and fruits, could lead to reward deprivation, potentially limiting the mood-improving effects of KD (Mela et al., 2026). Furthermore, restriction of other nutrients, including high-quality protein, may also reduce serotonin synthesis, thus affecting the improvement of depressive symptoms (Brinkworth et al., 2016).

**Table 1:** Summary of clinical interventional studies on the effects of different diet therapies on depressive symptoms in obese patients.

Diet therapy	Population	Length of intervention	Changes in depressive symptoms (mean ± SD or mean; 95%CI)	Notes and references
Mediterranean diet and ketogenic diet	Mediterranean diet *n* = 23; ketogenic diet *n* = 14; Aged 18–65, BMI 30–45 kg/m^2^; Spain	3 months	Change in BDI score: baseline Med:14.55 ± 6.32; Keto:13.83 ± 10.50. Less improvement with the ketogenic diet compared to the mediterranean diet (*p* < 0.05).	Figure only, original data unavailable (Mela et al., 2026)
MIND diet and CRD	MIND diet *n* = 22; CRD *n* = 22; Average age 55.36 and 54.68, BMI 30.94 and 31.31 kg/m^2^; Iran	12 weeks	MIND diet group and low-calorie diet group DASS depression score: Baseline 9.91 ± 10.98, 12.73 ± 9.96; End of trial 6.27 ± 7.64, 10.27 ± 8.10; *p* = 0.03.	Golmohammadi et al. (2025)
Probiotic supplement CRD and CRD	Probiotic supplement CRD *n* = 13; CRD *n* = 12; Aged 25–44, BMI 30.0–39.99 kg/m^2^, all male; Brazil	12 weeks	Probiotic supplement CRD group and CRD group BDI II score: Baseline 10.5(6.56–14.4), 9.17(3.82–14.5); 12 week 3.54(1.47–5.61), 5.82(1.42–10.2). *p* < 0.001; η^2^ρ = 0.448. DASS-21 score: 10.3(4.56–16.1), 7.50(1.74–13.3); 12 week 4.77(1.40–8.14), 4.73(0.68–8.77). *p* = 0.003; η^2^ρ = 0.345.	Marques et al. (2025)
Moderately high-protein diet and low-fat diet	Moderately high-protein diet *n* = 146; Low-fat diet *n* = 159; Average age 44.8 and 45.8, BMI 31.4 and 31.8 kg/m^2^, female 71.0%; Spain	16 weeks	Moderately High-protein diet & Low-fat diet **Δ**BDI score: −2.8 (−2.0; −3.6), −2.5 (−1.8; −3.2). *β* = 0.22, *p* = 0.04.	Rodriguez-Lozada et al. (2019)
VLCD and HCD	VLCD *n* = 41; HCD *n* = 37; Aged 58.5 ± 7.1, BMI 34.6 ± 4.3 kg/m^2^; Australia	52 weeks	Change in BDI score: significant improvements occurred while there was no statistically significant difference between the two groups (time × diet interaction, *p* ≥ 0.22).	Both groups underwent the same calorie restriction level, figure only, original data unavailable (Brinkworth et al., 2016)
LPHC and HPLC	LPHC *n* = 14; HPLC *n* = 14; Aged 32 ± 1.2 and 33 ± 1.2, overweight female with polycystic ovary syndrome; Australia	16 weeks	Change in HADS-depression score: significant improvement was observed only in the HPLC group.	Original data unavailable (Galletly et al., 2007)

BMI, Body Mass Index; BDI, Beck Depression Inventory; MIND, Mediterranean diet and Dietary Approaches to Stop Hypertension diet; CRD, Calorie Restriction Diet; DASS, Depression Anxiety Stress Scales; VLCD, Very Low-carbohydrate, High-fat Diet; HCD, High-carbohydrate, Low-fat Diet; LPHC, Low-protein High-carbohydrate Diet; HPLC, High-protein Low-carbohydrate diet; HADS, The Hospital Anxiety and Depression Scale.

## 4 Discussion

The evidence reviewed herein suggests that specific dietary patterns may simultaneously ameliorate both obesity and depressive symptoms by modulating neural, endocrine, and immune networks. These mechanisms are complex and diverse, including inflammatory pathways, neuroplasticity and neurotrophic factors, the gut-brain axis, the HPA axis, improved insulin sensitivity and brain energy metabolism, epigenetic modifications, oxidative stress levels, and metabolic homeostasis (Figure 2). Therefore, diet therapy, as a systemic treatment strategy that can reprogram the metabolic-psychological coupling to regulate obesity and depressive symptoms, has unique advantages and potential.

Based on the existing clinical evidence reviewed in this study, the Mediterranean and MIND diets appear to be more effective in improving depressive symptoms in obese patients, while the effects of diets like KD may be less pronounced. Therefore, in clinical practice, physicians may need to tailor dietary plans to patients with varying mental health states. This implementation of precise nutrition contributes to the development and widespread adoption of personalized medicine. Data comparisons among different diet therapies, such as intermittent diets, plant-based diets, and low-GI diets, remain largely unavailable, requiring further research.

In the preceding section, our study has examined various clinically commonly used dietary strategies that have demonstrated dual benefits for weight management and mood improvement in clinical trials. These effects are underpinned by a complex array of complementary mechanisms, including reducing systemic inflammation, increasing neurotrophic factor levels, regulating gut microbiota composition, modulating the HPA axis, and improving insulin sensitivity. These pathways rely on various substances, including SCFAs, omega-3 fatty acids, vitamin D, vitamin E, zinc, magnesium, among others (Schneider et al., 2024; Xiao Y. L. et al., 2024). The diversity and complexity of dietary intake, coupled with the interactions and multiple effects of these substances, makes their studies challenging. Furthermore, interventions targeting eating behavior itself, such as time-restricted diets, highlight the spatiotemporal heterogeneity of diets, further complicating the exploration of the fundamental principles behind diet therapy.

The available evidence suggests that diet therapy may represent a promising strategy for alleviating depressive symptoms in obese individuals. Future research should aim to elevate diet therapy from lifestyle recommendations to evidence-based metabolic-psychiatric interventions. Therefore, we believe further analysis based on genomics, metabolomics, microbiome, and epigenomics is needed to provide personalized and precise nutritional recommendations. Meanwhile, the development and widespread adoption of app-based personalized digital health tools, such as online support systems for remote monitoring of dietary interventions, may improve long-term patient adherence. Furthermore, exploring novel dietary interventions is equally important; cyclical ketogenic diets, specific amino acid restriction diets, and time-restricted dietary interventions still require further exploration and optimization. Future research should utilize neuroimaging, cerebrospinal fluid analysis, and molecular biology techniques to elucidate the mechanisms by which diet simultaneously affects metabolism and mood, drawing on temporal and spatial data. Equally important, researchers should explore how to ensure the correct implementation of diet therapies under limited time, material resources, and human resources.

Nevertheless, our present review has several limitations that should be acknowledged. First, existing research exhibits varying degrees of heterogeneity in dietary intervention methods, duration, control design, and outcome measurement, limiting the comparability of conclusions. Furthermore, most dietary intervention studies are short-term, typically less than 6 months, lacking long-term effects and maintenance data. And unfortunately, the original data of some existing research is unavailable. While researchers have proposed various mechanistic hypotheses, much direct human clinical evidence and intermediate molecular biological mechanisms remain missing (O'Hara and Gibney, 2021). The limited number of studies comparing the efficacy of different diet therapies may affect our assessment of their true effectiveness.

## 5 Conclusion

Accumulating evidence from the studies reviewed herein suggests that dietary interventions can improve both metabolic parameters and mood by targeting shared pathophysiological pathways involving immunity, metabolism, and neural function. This review also provides a comparative analysis of the clinical efficacy of various diet therapies in improving depressive symptoms in obese patients, reflecting on the actual mechanisms of action of these therapies from a clinical efficacy perspective. Clinicians can refer to this existing evidence to develop and adjust dietary plans for the mental health of obese patients. Future research should further elucidate the numerous molecular mechanisms that remain incompletely characterized. Current evidence suggests that diet therapy has the potential to become an important component of future precision medicine, promoting a shift in the control of obesity and depressive symptoms towards a more integrated, personalized, and prevention-oriented model. At the same time, therapy of this kind also empowers patients with the means and ability to manage their own health, which has profound clinical and public health significance. However, the molecular mechanisms of action of diet therapies on metabolism and mental health still requires robust validation. Future research should prioritize large-scale, multicenter, prospective RCTs to make a multidimensional assessment of the efficacy of dietary therapy for depressive symptoms in obese patients and the differences in efficacy among different diet therapies. Network analyses and well-designed individual studies examining the effects of specific dietary components on mental health outcomes are equally warranted.

## References

[ref1] Abdel-Rahman M. Hussein A. A. Ahmed-Farid O. A. Sawi A. A. Abdel Moneim A. E. (2024). Intermittent fasting alerts neurotransmitters and oxidant/antioxidant status in the brain of rats. Metab. Brain Dis. 39, 1291–1305. doi: 10.1007/s11011-024-01415-7, 39292431 PMC11513736

[ref2] Abiri B. Sarbakhsh P. Vafa M. (2022). Randomized study of the effects of vitamin D and/or magnesium supplementation on mood, serum levels of BDNF, inflammation, and SIRT1 in obese women with mild to moderate depressive symptoms. Nutr. Neurosci. 25, 2123–2135. doi: 10.1080/1028415X.2021.1945859, 34210242

[ref3] Adan R. A. H. van der Beek E. M. Buitelaar J. K. Cryan J. F. Hebebrand J. Higgs S. . (2019). Nutritional psychiatry: towards improving mental health by what you eat. Eur. Neuropsychopharmacol. 29, 1321–1332. doi: 10.1016/j.euroneuro.2019.10.011, 31735529

[ref4] Adolph T. E. Tilg H. (2024). Western diets and chronic diseases. Nat. Med. 30, 2133–2147. doi: 10.1038/s41591-024-03165-6, 39085420

[ref5] Akhondzadeh S. Mostafavi S. A. Keshavarz S. A. Mohammadi M. R. Hosseini S. Eshraghian M. R. (2020). A placebo controlled randomized clinical trial of *Crocus sativus* L. (saffron) on depression and food craving among overweight women with mild to moderate depression. J. Clin. Pharm. Ther. 45, 134–143. doi: 10.1111/jcpt.13040, 31602695

[ref6] Aleksandrova K. Koelman L. Rodrigues C. E. (2021). Dietary patterns and biomarkers of oxidative stress and inflammation: a systematic review of observational and intervention studies. Redox Biol. 42:101869. doi: 10.1016/j.redox.2021.101869, 33541846 PMC8113044

[ref7] Amadio P. Macchi C. Favero C. Zarà M. Solazzo G. Dioni L. . (2022). Brain-derived neurotrophic factor and extracellular vesicle-derived miRNAs in an Italian cohort of individuals with obesity: a key to explain the link between depression and atherothrombosis. Front. Cardiovasc. Med. 9:906483. doi: 10.3389/fcvm.2022.906483, 35911513 PMC9326054

[ref8] Applewhite B. Penninx B. Young A. H. Schmidt U. Himmerich H. Keeler J. L. (2024). The effect of a low-calorie diet on depressive symptoms in individuals with overweight or obesity: a systematic review and meta-analysis of interventional studies. Psychol. Med. 54, 1671–1683. doi: 10.1017/S0033291723003586, 38084632

[ref9] Arab A. Khorvash F. Kazemi M. Heidari Z. Askari G. (2022). Effects of the dietary approaches to stop hypertension (DASH) diet on clinical, quality of life and mental health outcomes in women with migraine: a randomised controlled trial. Br. J. Nutr. 128, 1535–1544. doi: 10.1017/S000711452100444X, 34763733

[ref10] Baek J. H. Zhu Y. Jackson C. L. Park Y. M. (2024). Artificial light at night and type 2 diabetes mellitus. Diabetes Metab. J. 48, 847–863. doi: 10.4093/dmj.2024.0237, 39313230 PMC11449813

[ref11] Bahadorpour S. Hajhashemy Z. Mohammadi S. Mokhtari E. Heidari Z. Saneei P. (2023). Total fat and omega-3 fatty acids intake in relation to serum brain-derived neurotrophic factor (BDNF) levels and psychological disorders in Iranian adults. Sci. Rep. 13:5392. doi: 10.1038/s41598-023-32510-x, 37012351 PMC10068220

[ref12] Bansal S. Agrawal M. Mahendiratta S. Kumar S. Arora S. Joshi R. . (2021). Everolimus: a potential therapeutic agent targeting PI3K/Akt pathway in brain insulin system dysfunction and associated neurobehavioral deficits. Fundam. Clin. Pharmacol. 35, 1018–1031. doi: 10.1111/fcp.12677, 33783880

[ref13] Becker F. Behrends M. M. Rudolph K. L. (2023). Evolution, mechanism and limits of dietary restriction induced health benefits & longevity. Redox Biol. 63:102725. doi: 10.1016/j.redox.2023.102725, 37257276 PMC10245103

[ref14] Bennell K. L. Lawford B. J. Keating C. Brown C. Kasza J. Mackenzie D. . (2022). Comparing video-based, telehealth-delivered exercise and weight loss programs with online education on outcomes of knee osteoarthritis: a randomized trial. Ann. Intern. Med. 175, 198–209. doi: 10.7326/M21-2388, 34843383

[ref15] Blasco B. V. García-Jiménez J. Bodoano I. Gutiérrez-Rojas L. (2020). Obesity and depression: its prevalence and influence as a prognostic factor: a systematic review. Psychiatry Investig. 17, 715–724. doi: 10.30773/pi.2020.0099, 32777922 PMC7449839

[ref16] Breymeyer K. L. Lampe J. W. McGregor B. A. Neuhouser M. L. (2016). Subjective mood and energy levels of healthy weight and overweight/obese healthy adults on high-and low-glycemic load experimental diets. Appetite 107, 253–259. doi: 10.1016/j.appet.2016.08.008, 27507131 PMC5154680

[ref17] Brinkworth G. D. Luscombe-Marsh N. D. Thompson C. H. Noakes M. Buckley J. D. Wittert G. . (2016). Long-term effects of very low-carbohydrate and high-carbohydrate weight-loss diets on psychological health in obese adults with type 2 diabetes: randomized controlled trial. J. Intern. Med. 280, 388–397. doi: 10.1111/joim.12501, 27010424

[ref18] Cao J. Chen Z. Wang Y. Ma Y. Yang Z. Cai J. . (2024). Overweight and glucose/lipid metabolism abnormality associated with SSRIs: a pharmacovigilance study based on the FDA adverse event reporting system. Front. Pharmacol. 15:1517546. doi: 10.3389/fphar.2024.1517546, 39867657 PMC11759304

[ref19] Chen S. M. Hsiao C. W. Chen Y. J. Hong C. J. Lin J. C. Yang C. P. . (2024). Interleukin-4 inhibits the hypothalamic appetite control by modulating the insulin-AKT and JAK-STAT signaling in leptin mutant mice. Environ. Toxicol. 39, 3980–3990. doi: 10.1002/tox.24264, 38597583

[ref20] Chrysafi M. Jacovides C. Papadopoulou S. K. Psara E. Vorvolakos T. Antonopoulou M. . (2024). The potential effects of the ketogenic diet in the prevention and co-treatment of stress, anxiety, depression, schizophrenia, and bipolar disorder: from the basic research to the clinical practice. Nutrients 16. doi: 10.3390/nu16111546, 38892480 PMC11174630

[ref21] Daneshzad E. Heshmati J. Basirat V. Keshavarz S. A. Qorbani M. Larijani B. . (2022). The effect of the dietary approaches to stop hypertension (DASH) diet on sleep, mental health, and hormonal changes: a randomized clinical trial in women with type 2 diabetes. Front. Nutr. 9:775543. doi: 10.3389/fnut.2022.775543, 35634394 PMC9133887

[ref22] Depboylu G. Y. Bildik O. Kaner G. Gençpınar P. Dündar N. O. (2025). The effects of low glycemic index diet on epileptic seizure frequency, oxidative stress, mental health, and health-related quality of life in children with drug-resistant epilepsy. Seizure 127, 57–65. doi: 10.1016/j.seizure.2025.03.010, 40112488

[ref23] Ding H. Ouyang M. Wang J. Xie M. Huang Y. Yuan F. . (2022). Shared genetics between classes of obesity and psychiatric disorders: a large-scale genome-wide cross-trait analysis. J. Psychosom. Res. 162:111032. doi: 10.1016/j.jpsychores.2022.111032, 36137488

[ref24] Dong Y. Cui C. (2022). The role of short-chain fatty acids in central nervous system diseases. Mol. Cell. Biochem. 477, 2595–2607. doi: 10.1007/s11010-022-04471-8, 35596843

[ref25] Ferreira-Hermosillo A. de Miguel Ibañez R. Pérez-Dionisio E. K. Villalobos-Mata K. A. (2023). Obesity as a neuroendocrine disorder. Arch. Med. Res. 54:102896. doi: 10.1016/j.arcmed.2023.102896, 37945442

[ref26] Galletly C. Moran L. Noakes M. Clifton P. Tomlinson L. Norman R. (2007). Psychological benefits of a high-protein, low-carbohydrate diet in obese women with polycystic ovary syndrome-a pilot study. Appetite 49, 590–593. doi: 10.1016/j.appet.2007.03.222, 17509728

[ref27] GBD 2019 Diseases and Injuries Collaborators (2020). Global burden of 369 diseases and injuries in 204 countries and territories, 1990-2019: a systematic analysis for the global burden of disease study 2019. Lancet 396, 1204–1222. doi: 10.1016/S0140-6736(20)30925-9 33069326 PMC7567026

[ref28] Gianfredi V. Koster A. Odone A. Amerio A. Signorelli C. Schaper N. C. . (2021). Associations of dietary patterns with incident depression: the Maastricht study. Nutrients 13. doi: 10.3390/nu13031034, 33806882 PMC8004955

[ref29] Golmohammadi M. Attari V. E. Salimi Y. Saed L. Nachvak S. M. Samadi M. (2025). The effect of MIND diet on sleep status, mental health, and serum level of BDNF in overweight/obese diabetic women with insomnia: a randomized controlled trial. Sci. Rep. 15:8237. doi: 10.1038/s41598-025-91389-y, 40065021 PMC11893750

[ref30] Hall K. D. Ayuketah A. Brychta R. Cai H. Cassimatis T. Chen K. Y. . (2019). Ultra-processed diets cause excess calorie intake and weight gain: an inpatient randomized controlled trial of ad libitum food intake. Cell Metab. 30, 67–77.e3. doi: 10.1016/j.cmet.2019.05.008, 31105044 PMC7946062

[ref31] Hansen B. Roomp K. Ebid H. Schneider J. G. (2024). Perspective: the impact of fasting and caloric restriction on neurodegenerative diseases in humans. Adv. Nutr. 15:100197. doi: 10.1016/j.advnut.2024.100197, 38432589 PMC10997874

[ref32] Hodes G. E. Bangasser D. Sotiropoulos I. Kokras N. Dalla C. (2024). Sex differences in stress response: classical mechanisms and beyond. Curr. Neuropharmacol. 22, 475–494. doi: 10.2174/1570159X22666231005090134, 37855285 PMC10845083

[ref33] Huang X. Liang J. Zhang J. Fu J. Xie W. Zheng F. (2024). Association of cardiovascular-kidney-metabolic health and social connection with the risk of depression and anxiety. Psychol. Med. 54, 4203–4211. doi: 10.1017/S0033291724002381, 39552398 PMC11650165

[ref34] Imayama I. Alfano C. M. Kong A. Foster-Schubert K. E. Bain C. E. Xiao L. . (2011). Dietary weight loss and exercise interventions effects on quality of life in overweight/obese postmenopausal women: a randomized controlled trial. Int. J. Behav. Nutr. Phys. Act. 8:118. doi: 10.1186/1479-5868-8-118, 22026966 PMC3215656

[ref35] Ito M. Nishida Y. Iwamoto T. Kanai A. Aoyama S. Ueki K. . (2024). Protein acylations induced by a ketogenic diet demonstrate diverse patterns depending on organs and differ between histones and global proteins. Biochem. Biophys. Res. Commun. 712-713:149960. doi: 10.1016/j.bbrc.2024.149960, 38640734

[ref36] Jia M. Hu X. Ning F. Geng K. Yuan T. Liu Z. (2025). Dietary Fiber deprivation disrupts colonic homeostasis to drive anxiety-like behavior via the gut-brain Axis. J. Agric. Food Chem. 73, 32685–32699. doi: 10.1021/acs.jafc.5c12112, 41364932

[ref37] Keswani R. Thorat V. G. Patil C. R. Bhatt S. (2025). Exploring the role of adipocytokines in obesity associated depression. Drug Discov. Today 30:104509. doi: 10.1016/j.drudis.2025.104509, 41201620

[ref38] Khan M. S. Faizan M. Yang G. Kang K. S. (2026). Gut dysbiosis-mediated major depressive disorder: a review of pathogenic mechanisms and potential therapeutic strategies. Cells 15. doi: 10.3390/cells15110972, 42274565 PMC13256297

[ref39] Khavandegar A. Heidarzadeh A. Angoorani P. Hasani-Ranjbar S. Ejtahed H. S. Larijani B. . (2024). Adherence to the Mediterranean diet can beneficially affect the gut microbiota composition: a systematic review. BMC Med. Genet. 17:91. doi: 10.1186/s12920-024-01861-3, 38632620 PMC11022496

[ref40] Li R. J. Liu Y. Liu H. Q. Li J. (2020). Ketogenic diets and protective mechanisms in epilepsy, metabolic disorders, cancer, neuronal loss, and muscle and nerve degeneration. J. Food Biochem. 44:e13140. doi: 10.1111/jfbc.13140, 31943235

[ref41] Liang J. Zhang J. Sun J. Liang Q. Zhan Y. Yang Z. . (2025). Ketogenic diet attenuates neuroinflammation and restores hippocampal neurogenesis to improve CUMS induced depression-like behavior in mice. Food Funct. 16, 3408–3422. doi: 10.1039/D5FO00226E, 40197680

[ref42] Lincoff A. M. Brown-Frandsen K. Colhoun H. M. Deanfield J. Emerson S. S. Esbjerg S. . (2023). Semaglutide and cardiovascular outcomes in obesity without diabetes. N. Engl. J. Med. 389, 2221–2232. doi: 10.1056/NEJMoa2307563, 37952131

[ref43] Lingvay I. Cohen R. V. Roux C. W. L. Sumithran P. (2024). Obesity in adults. Lancet 404, 972–987. doi: 10.1016/S0140-6736(24)01210-8, 39159652

[ref44] Lof J. Smits K. Melotte V. Kuil L. E. (2022). The health effect of probiotics on high-fat diet-induced cognitive impairment, depression and anxiety: a cross-species systematic review. Neurosci. Biobehav. Rev. 136:104634. doi: 10.1016/j.neubiorev.2022.104634, 35339484

[ref45] Luppino F. S. de Wit L. M. Bouvy P. F. Stijnen T. Cuijpers P. Penninx B. W. . (2010). Overweight, obesity, and depression: a systematic review and meta-analysis of longitudinal studies. Arch. Gen. Psychiatry 67, 220–229. doi: 10.1001/archgenpsychiatry.2010.2, 20194822

[ref46] Lv N. Kringle E. A. Ma J. (2022). Integrated Behavioral interventions for adults with comorbid obesity and depression: a systematic review. Curr. Diab. Rep. 22, 157–168. doi: 10.1007/s11892-022-01458-z, 35304703 PMC8930483

[ref47] Marques C. G. Dos Santos Quaresma M. V. L. França Ferracini C. B. Alves Carrilho F. B. Nakamoto F. P. Lucin G. A. . (2025). Effect of caloric restriction with probiotic supplementation on body composition, quality of life, and psychobiological factors of obese men: a randomized, double-blinded placebo-controlled clinical trial. Clin. Nutr. 45, 234–249. doi: 10.1016/j.clnu.2024.12.031, 39842252

[ref48] McLachlan C. Shelton R. Li L. (2023). Obesity, inflammation, and depression in adolescents. Front. Psych. 14:1221709. doi: 10.3389/fpsyt.2023.1221709, 37840796 PMC10568138

[ref49] Mela V. Ortiz Samur N. S. Vijaya A. K. Gálvez V. J. García-Martín M. L. Bandera B. . (2026). Ketogenic diet is less effective in ameliorating depression and anxiety in obesity than Mediterranean diet: a pilot study for exploring the GUT-brain axis. Brain Behav. Immun. 131:106167. doi: 10.1016/j.bbi.2025.106167, 41197688

[ref50] Menniti G. Meshkat S. Lin Q. Lou W. Reichelt A. Bhat V. (2025). Mental health consequences of dietary restriction: increased depressive symptoms in biological men and populations with elevated BMI. BMJ Nutr. Prev. Health 8:e001167. doi: 10.1136/bmjnph-2025-001167, 40771520 PMC12322571

[ref51] Millar S. R. Perry I. J. Phillips C. M. (2026). Plant-based dietary indices and mental health: a cross-sectional study of a middle- to older-aged population. Eur. J. Nutr. 65. doi: 10.1007/s00394-026-04000-z, 42191962 PMC13212728

[ref52] Miller A. H. Raison C. L. (2016). The role of inflammation in depression: from evolutionary imperative to modern treatment target. Nat. Rev. Immunol. 16, 22–34. doi: 10.1038/nri.2015.5, 26711676 PMC5542678

[ref53] Miranda A. M. A. McAllan L. Mazzei G. Andrew I. Davies I. Ertugrul M. . (2025). Selective remodelling of the adipose niche in obesity and weight loss. Nature 644, 769–779. doi: 10.1038/s41586-025-09233-2, 40634602 PMC12367556

[ref54] Mostafavi Abdolmaleky H. Pirani A. Pettinato G. (2025). Psychosomatic disorders, epigenome, and gut microbiota. Cells 14. doi: 10.3390/cells14241959, 41439979 PMC12732280

[ref55] Nagi M. A. Ahmed H. Rezq M. A. A. Sangroongruangsri S. Chaikledkaew U. Almalki Z. . (2024). Economic costs of obesity: a systematic review. Int. J. Obes. 48, 33–43. doi: 10.1038/s41366-023-01398-y, 37884664

[ref56] Niu H. Zhou M. Zogona D. Xing Z. Wu T. Chen R. . (2024). *Akkermansia muciniphila*: a potential candidate for ameliorating metabolic diseases. Front. Immunol. 15:1370658. doi: 10.3389/fimmu.2024.1370658, 38571945 PMC10987721

[ref57] O'Hara C. Gibney E. R. (2021). Meal pattern analysis in nutritional science: recent methods and findings. Adv. Nutr. 12, 1365–1378. doi: 10.1093/advances/nmaa175, 33460431 PMC8321870

[ref58] Ohtsuki S. (2024). Insulin receptor at the blood-brain barrier: transport and signaling. Vitam. Horm. 126, 113–124. doi: 10.1016/bs.vh.2024.05.001, 39029970

[ref59] Oracz A. J. Zwierz M. Naumowicz M. Suprunowicz M. Waszkiewicz N. (2025). Relationship between obesity and depression considering the inflammatory theory. Int. J. Mol. Sci. 26. doi: 10.3390/ijms26114966, 40507778 PMC12154410

[ref60] Pang Z. P. Han W. (2012). Regulation of synaptic functions in central nervous system by endocrine hormones and the maintenance of energy homoeostasis. Biosci. Rep. 32, 423–432. doi: 10.1042/BSR20120026, 22582733 PMC3804927

[ref61] Paris T. Daly R. M. Abbott G. Sood S. Freer C. L. Ryan M. C. . (2024). Diet overall and hypocaloric diets are associated with improvements in depression but not anxiety in people with metabolic conditions: a systematic review and Meta-analysis. Adv. Nutr. 15:100169. doi: 10.1016/j.advnut.2024.100169, 38184198 PMC10847486

[ref62] Pavithran N. Kumar H. Menon A. S. Pillai G. K. Sundaram K. R. Ojo O. (2020). The effect of a low GI diet on truncal fat mass and glycated Hemoglobin in south Indians with type 2 diabetes-a single Centre randomized prospective study. Nutrients 12. doi: 10.3390/nu12010179, 31936428 PMC7019781

[ref63] Reagan L. P. Cowan H. B. Woodruff J. L. Piroli G. G. Erichsen J. M. Evans A. N. . (2021). Hippocampal-specific insulin resistance elicits behavioral despair and hippocampal dendritic atrophy. Neurobiol. Stress 15:100354. doi: 10.1016/j.ynstr.2021.100354, 34258333 PMC8252121

[ref64] Rodriguez-Lozada C. Cuervo M. Cuevas-Sierra A. Goni L. Riezu-Boj J. I. Navas-Carretero S. . (2019). Changes in anxiety and depression traits induced by energy restriction: predictive value of the baseline status. Nutrients 11. doi: 10.3390/nu11061206, 31141954 PMC6627283

[ref65] Rondanelli M. Opizzi A. Faliva M. Bucci M. Perna S. (2012). Relationship between the absorption of 5-hydroxytryptophan from an integrated diet, by means of Griffonia simplicifolia extract, and the effect on satiety in overweight females after oral spray administration. Eat. Weight Disord. 17, e22–e28. doi: 10.3275/8165, 22142813

[ref66] Scherer T. Sakamoto K. Buettner C. (2021). Brain insulin signalling in metabolic homeostasis and disease. Nat. Rev. Endocrinol. 17, 468–483. doi: 10.1038/s41574-021-00498-x, 34108679

[ref67] Schneider E. O'Riordan K. J. Clarke G. Cryan J. F. (2024). Feeding gut microbes to nourish the brain: unravelling the diet-microbiota-gut-brain axis. Nat. Metab. 6, 1454–1478. doi: 10.1038/s42255-024-01108-6, 39174768

[ref68] Selman A. Dai J. Driskill J. Reddy A. P. Reddy P. H. (2025). Depression and obesity: focus on factors and mechanistic links. Biochim. Biophys. Acta Mol. Basis Dis. 1871:167561. doi: 10.1016/j.bbadis.2024.167561, 39505048

[ref69] Semnani-Azad Z. Khan T. A. Chiavaroli L. Chen V. Bhatt H. A. Chen A. . (2025). Intermittent fasting strategies and their effects on body weight and other cardiometabolic risk factors: systematic review and network meta-analysis of randomised clinical trials. BMJ 389:e082007. doi: 10.1136/bmj-2024-082007, 40533200 PMC12175170

[ref70] Son J. W. Kim S. (2020). Comprehensive review of current and upcoming anti-obesity drugs. Diabetes Metab. J. 44, 802–818. doi: 10.4093/dmj.2020.0258, 33389955 PMC7801751

[ref71] Stewart R. A. H. (2018). Primary prevention of cardiovascular disease with a Mediterranean diet supplemented with extra-virgin olive oil or nuts. N. Engl. J. Med. 379, 1387–1389. doi: 10.1056/NEJMc1809971, 30285332

[ref72] Tan S. Chen W. Kong G. Wei L. Xie Y. (2023). Peripheral inflammation and neurocognitive impairment: correlations, underlying mechanisms, and therapeutic implications. Front. Aging Neurosci. 15:1305790. doi: 10.3389/fnagi.2023.1305790, 38094503 PMC10716308

[ref73] Toh D. W. K. Koh E. S. Kim J. E. (2020). Lowering breakfast glycemic index and glycemic load attenuates postprandial glycemic response: a systematically searched meta-analysis of randomized controlled trials. Nutrition 71:110634. doi: 10.1016/j.nut.2019.110634, 31838460

[ref74] van Heesch F. Prins J. Korte-Bouws G. A. Westphal K. G. Lemstra S. Olivier B. . (2013). Systemic tumor necrosis factor-alpha decreases brain stimulation reward and increases metabolites of serotonin and dopamine in the nucleus accumbens of mice. Behav. Brain Res. 253, 191–195. doi: 10.1016/j.bbr.2013.07.038, 23896053

[ref75] Varady K. A. Cienfuegos S. Ezpeleta M. Gabel K. (2021). Cardiometabolic benefits of intermittent fasting. Annu. Rev. Nutr. 41, 333–361. doi: 10.1146/annurev-nutr-052020-041327, 34633860

[ref76] Wang J. Wang F. Chen H. Liu L. Zhang S. Luo W. . (2022). Comparison of the effects of intermittent energy restriction and continuous energy restriction among adults with overweight or obesity: an overview of systematic reviews and Meta-analyses. Nutrients 14. doi: 10.3390/nu14112315, 35684119 PMC9183159

[ref77] Wang X. Wang X. Yu S. Huang L. Xue Q. Yang X. . (2025). Schisantherin a interacts with gut bacteria to stimulate adipose tissue thermogenesis in obese mice via a TGR5–p-CREB–STAT6 signaling pathway. Nat. Commun. 17:476. doi: 10.1038/s41467-025-67172-y, 41381461 PMC12800289

[ref78] Wang X. Yin Z. Yang Y. Fu X. Guo C. Pu K. . (2024). Association of plant-based dietary patterns with depression and anxiety symptoms in Chinese older adults: a nationwide study. J. Affect. Disord. 350, 838–846. doi: 10.1016/j.jad.2024.01.159, 38278327

[ref79] Waśkiewicz Z. (2026). Carnivore and animal-based diets in sport: a critical evaluation of current evidence and future perspectives for precision nutrition. Nutrients 18. doi: 10.3390/nu18060998, 41901173 PMC13029088

[ref80] Xiao Y. L. Gong Y. Qi Y. J. Shao Z. M. Jiang Y. Z. (2024). Effects of dietary intervention on human diseases: molecular mechanisms and therapeutic potential. Signal Transduct. Target. Ther. 9:59. doi: 10.1038/s41392-024-01771-x, 38462638 PMC10925609

[ref81] Xiao Y. Xiao X. Zhang X. Yi D. Li T. Hao Q. . (2024). Mediterranean diet in the targeted prevention and personalized treatment of chronic diseases: evidence, potential mechanisms, and prospects. EPMA J. 15, 207–220. doi: 10.1007/s13167-024-00360-w, 38841625 PMC11147989

[ref82] Xie X. Zhang M. Luo H. (2024). Regulation of metabolism by circadian rhythms: support from time-restricted eating, intestinal microbiota & omics analysis. Life Sci. 351:122814. doi: 10.1016/j.lfs.2024.122814, 38857654

[ref83] Xu X. Piao H. N. Aosai F. Zeng X. Y. Cheng J. H. Cui Y. X. . (2020). Arctigenin protects against depression by inhibiting microglial activation and neuroinflammation via HMGB1/TLR4/NF-κB and TNF-α/TNFR1/NF-κB pathways. Br. J. Pharmacol. 177, 5224–5245. doi: 10.1111/bph.15261, 32964428 PMC7589024

[ref84] Xu J. Xie L. Fan R. Shi X. Xu W. Dong K. . (2025). The role of dietary inflammatory index in metabolic diseases: the associations, mechanisms, and treatments. Eur. J. Clin. Nutr. 79, 397–412. doi: 10.1038/s41430-024-01525-6, 39433856

[ref85] Yamada N. Katsuura G. Ochi Y. Ebihara K. Kusakabe T. Hosoda K. . (2011). Impaired CNS leptin action is implicated in depression associated with obesity. Endocrinology 152, 2634–2643. doi: 10.1210/en.2011-0004, 21521746

[ref86] Yildirim Z. Sanlier N. (2024). The relationship of certain diseases and dietary inflammatory index in older adults: a narrative review. Curr. Nutr. Rep. 13, 768–785. doi: 10.1007/s13668-024-00566-4, 39230632

[ref87] Yuan L. Su Y. Zhao J. Cho M. Wang D. Yuan L. . (2025). Investigating the shared genetic architecture between obesity and depression: a large-scale genomewide cross-trait analysis. Front. Endocrinol. (Lausanne) 16:1578944. doi: 10.3389/fendo.2025.1578944, 40405979 PMC12094978

[ref88] Zamani B. Daneshzad E. Siassi F. Guilani B. Bellissimo N. Azadbakht L. (2020). Association of plant-based dietary patterns with psychological profile and obesity in Iranian women. Clin. Nutr. 39, 1799–1808. doi: 10.1016/j.clnu.2019.07.019, 31399262

[ref89] Zhang W. Chen P. Huo S. Huang X. Zhao Y. (2024). Requirements for essential micronutrients during caloric restriction and fasting. Front. Nutr. 11:1363181. doi: 10.3389/fnut.2024.1363181, 38481969 PMC10936542

